# Conditions Affected by Storms

**Published:** 1896-04

**Authors:** C. L. Olds


### CONDITIONS AFFECTED BY STORMS.

EDITOR OF THE HOMŒOPATHIC PHYSICIAN :

In the February issue of your journal, page 101, I read with much interest an article entitled " Conditions Affected by Storms." But knowing of several remedies, not mentioned by the author, that would come under this head, I examined several repertories and works on materia medica, the result of which I thought might be of interest to the readers of THE HOMŒOPATHIC PHYSICIAN.

In order that this may be more complete, I will also introduce the remedies given by the author of the above-mentioned article.

In the *Guiding Symptoms* we may find the following :

Worse before a thunder-storm. Agar.

Stormy weather ; oppressed breathing. Ars.

Before a thunder-storm, many old symptoms appear again. Ars-hyd.

Worse in stormy weather. Bad.

Stormy change of weather; facial neuralgia. Caust.

Worse before a storm; neuralgic pains. Cedron.

On approach of rain, pain in nose worse. Hep.

Attacks return before storms. Hyper.

Stormy weather; anxiety. Lyc.

Approach of rain-storm or in rainy weather; rheumatic pains in joints. Melilotus.

During storm flatulent colic, worse. Natr-sul.

During thunder-storm, trembling and palpitation, pains worse. Natr-phos.

Aggravation before and during thunder-storm. Petr.

During thunder-storm, anxious. Phos.

Before thunder-storm, cough. Phos.

Aggravation before thunder-storm. Puls.

Aggravation before storm; inflammatory rheumatism. Rhus.

Aggravation before and *during* storm. Rhus. (*Italics mine.*)

Aggravation *before* and during storm. Rhod. (*Italics mine.*)

Aggravation, stormy weather. Sep.

Before a storm, ciliary neuralgia. Sil.

Aggravation during thunder-storm. Sil.

Before storm, ulcer on leg worse. Sulph.

During thunder-storm; bronchial asthma worse. Syph.

Approach of storms; chills. Zn.

Dr. A. Lippe, in his *Comparative Materia Medica* gives: Aggravation during a thunder-storm. Bry., Lach., Natr-carb., Rhod., Sil.

Dr. Lee, in his *Repertory of the Symptoms of the Mind*, gives: Anxiety during a storm. Gels., *Natr-carb.*, Natr-mur., *Nitr-ac.*, Phos.

In Bœnninghausen's *Therapeutic Pocket Book*, Allen gives: Aggravation on approach of a storm. *Bry.*, *Hyper.*, *Meli.*, *RHODO.*

Aggravation during thunder-storm. *Bry.*, Caust., *Lach.*, NAT-C., Nat-m., Nit-ac., Petrol., *Phos.*, RHODO., SIL.

Worcester in his *Repertory to the Modalities*," gives under the heading, "Effects of Thunder-storms," the following remedies: Agar., Gels., Hyper., Nat-c., Nux-m., Petr., Phos., Psor., Puls., Rhod., Sil., Zn.

In Lippe's *Repertory* we have:

Aggravation just before a thunder-storm. Agar., Lach., Natr-c., Phos., Rhod., Sil.

Aggravation during a thunder-storm. Bry., Caust., Lach., Natr-c., Natr-m., Nitr-ac., Petr., Phos., *Rhod.*, Sil. Thuj.

To summarize, we have:

Effects of storms. *Agar.*, Ars., Ars-hyd., *Bad.*, *Bry.*, Caust., Cedron, *Gels.*, Hep., *Hyper.*, *Lach.*, Lyc., Meli., *Natr-c.*, Natr-m., Natr-phos., Natr-sul., Nitr-ac., Nux-m., *Petr.*, *Phos.*, Psor., *Puls.*, *Rhus*, *Rhod.*, Sep., *Sil.*, Sulph., Syph., Thuj., Zn.

Aggravation before a storm. *Agar.*, Ars-hyd., *Bry.*, Cedron, Hep., *Hyper.*, Lach., Meli., Natr-c., Nux-m., Petr., Phos., Puls., Rhus, *Rhod.*, Sil., Sulph., Zn.

Aggravation during a storm. Ars., *Bad.*, Bry., Caust., Gels., *Lach.*, Lyc., Meli., Natr-c., Natr-m., Natr-phos., Natr-sul., Nitr-ac., Petr., *Phos.*, *Rhus*, Rhod., Sep., Sil., Syph., Thuj.

Aggravation before a thunder-storm. Agar., Ars-hyd., Gels., Lach., Natr-c., *Petr.*, Phos., Psor., *Puls.*, Rhus, *Rhod.*, Sil.

Aggravation during a thunder-storm. Agar., Bry., Caust., Lach., *Natr-c.*, Natr-m., Natr-phos., Nitr-ac., *Petr.*, *Phos.*, Psor., Rhus, Rhod., Sil., Syph., Thuj.

C. L. Olds.

